# Sensor-Based Assessment of Trunk–Lower Limb Neuromuscular Coordination During Flat Tennis Serve Using Surface Electromyography and Time–Frequency Intermuscular Coherence Analysis

**DOI:** 10.3390/s26216899

**Published:** 2026-10-31

**Authors:** Yifei Jiang, Weihao Jiang

**Affiliations:** School of Physical Education, Hangzhou Normal University, Hangzhou 311121, China; jiang_yifei0319@163.com

**Keywords:** tennis performance, neuromuscular control, surface electromyography, time–frequency analysis, skill level

## Abstract

Objective: To compare flat-serve performance, muscle activation, and trunk–lower limb time–frequency intermuscular coherence between professional and amateur tennis players. Methods: Twenty-four tennis players (17 male and 7 female), including 12 professional and 12 amateur players, participated in this cross-sectional group-comparison study. Each participant completed 10 valid flat serves under standardized testing conditions, and serve-placement accuracy was assessed using a predefined target area. Surface electromyographic signals from 10 dominant-side trunk and lower-limb muscles were recorded using a Delsys Trigno Wireless EMG System, while serve movements were captured using a Photron FASTCAM Mini AX200 high-speed camera. Electromyographic and video data were synchronized using a transistor–transistor logic trigger. Integrated electromyography (IEMG) was normalized to maximal voluntary contraction, and time–frequency intermuscular coherence was calculated for 21 trunk–lower limb muscle pairs across the α, β, and γ frequency bands. Between-group differences were examined using two-tailed independent-samples *t*-tests. The Benjamini–Hochberg false discovery rate (BH–FDR) procedure was applied to outcome families involving multiple comparisons, with statistical significance determined at FDR q < 0.05. Results: Serve-placement accuracy was significantly higher in the professional group than in the amateur group (69.2 ± 7.4% vs. 48.3 ± 8.9%; t = 6.26, raw *p* < 0.001, Cohen’s d = 2.55). After BH–FDR correction, professional players showed significantly higher IEMG values in the soleus (t = 3.12, raw *p* = 0.005, FDR q = 0.019, d = 1.27), vastus medialis (t = 2.92, raw *p* = 0.008, FDR q = 0.020, d = 1.19), vastus lateralis (t = 2.60, raw *p* = 0.016, FDR q = 0.027, d = 1.06), gluteus maximus (t = 3.17, raw *p* = 0.004, FDR q = 0.019, d = 1.29), and latissimus dorsi (t = 2.74, raw *p* = 0.012, FDR q = 0.024, d = 1.12), but significantly lower IEMG in the rectus abdominis (t = −3.07, raw *p* = 0.006, FDR q = 0.019, d = −1.25). Several muscle pair–frequency combinations showed nominal differences based on raw *p*-values and moderate-to-large effect sizes; however, none of the time–frequency intermuscular coherence comparisons remained statistically significant after BH–FDR correction (all FDR q > 0.05). Conclusion: Professional players demonstrated greater flat-serve placement accuracy and a distinct distribution of trunk and lower-limb muscle activation. The coherence findings were exploratory and did not conclusively establish skill-level differences in trunk–lower limb intermuscular coupling. Flat-serve training may emphasize lower-limb support and propulsion, hip extension, and trunk stabilization rather than focusing exclusively on arm or racket actions.

## 1. Introduction

The flat tennis serve is one of the most decisive technical skills in competitive tennis because it directly initiates the point and can create an immediate tactical advantage. Compared with rally-based strokes, the serve requires highly coordinated whole-body movement control and rapid force transmission within a very short time window [1]. In particular, the flat serve emphasizes ball speed, depth, and placement accuracy, making it an important scoring technique in high-level competition [2]. However, the complexity of the flat serve makes performance evaluation challenging because successful execution depends not only on upper-limb racket acceleration, but also on precise timing, postural control, and efficient coordination of the entire kinetic chain [3]. Therefore, objective sensing technologies capable of capturing neuromuscular characteristics during sport-specific movements are increasingly important for understanding performance-related mechanisms.

Recent advances in wearable sensing technologies have provided new opportunities for quantitative assessment of human movement and neuromuscular function during complex motor tasks. Among these technologies, surface electromyography (sEMG) sensors have been widely applied to monitor muscle activation patterns because they can provide non-invasive, real-time information regarding neuromuscular activity during dynamic movements. By collecting electrical signals generated by active muscles, sEMG-based sensing systems allow researchers to characterize muscle recruitment strategies, force-generation demands, and coordination patterns that cannot be fully identified through external performance outcomes alone [3]. In sports scenarios, wearable sEMG sensors combined with advanced signal-processing methods provide a valuable approach for investigating how athletes with different skill levels organize muscle activity during high-speed and technically demanding movements. Previous tennis research has also combined wearable electromyography, accelerometry, and vibration sensing to compare muscle activity and racket-related mechanical characteristics between experienced and recreational players under realistic playing conditions. These findings support the feasibility of applying synchronized wearable sensors to investigate competitive-level differences in tennis-specific neuromuscular function.

Although the racket is held by the upper limb, the tennis serve is not an isolated arm movement. Biomechanical studies have shown that effective serving relies on a proximal-to-distal kinetic chain, in which force is initiated through lower-limb loading and extension, transferred through the pelvis and trunk, and ultimately expressed through upper-limb and racket acceleration [4]. During the serve, knee-flexion loading contributes to mechanical energy storage, whereas subsequent hip–knee–ankle extension provides upward and forward propulsion [5]. The trunk functions as an essential mechanical and neuromuscular bridge by stabilizing the body and transferring force from the lower limbs to the striking arm [6]. Therefore, sensor-based evaluation focusing only on isolated upper-limb activity may provide an incomplete understanding of flat-serve performance, which depends on coordinated trunk–lower limb function and efficient kinetic-chain organization [7].

Although sEMG amplitude-based parameters, such as integrated electromyography (IEMG), can quantify overall muscle activation requirements during sport-specific movements, they cannot fully describe the temporal coordination and neural coupling between functionally related muscles [8]. To overcome this limitation, intermuscular coherence analysis has been introduced as a signal-processing approach to evaluate common oscillatory activity between muscles within specific frequency ranges, providing additional information regarding neuromuscular coupling beyond conventional EMG amplitude measurements [9]. Furthermore, time–frequency intermuscular coherence analysis enables characterization of dynamic changes in muscle coupling across both time and frequency domains, making it particularly suitable for rapid and phase-dependent skills such as tennis serving [10]. Previous studies have suggested that coherence within α-, β-, and γ-frequency bands may reflect different aspects of sensorimotor integration, corticospinal or supraspinal contributions, and rapid motor coordination demands during complex motor tasks [11]. Thus, combining wearable sEMG sensing with time–frequency signal analysis may provide a more comprehensive framework for evaluating skill-dependent neuromuscular coordination.

Participant characteristics should also be considered when interpreting sensor-derived neuromuscular measurements. Previous tennis research has included both male and female collegiate players in EMG-based assessments of serve and forehand performance and found that sex did not significantly modify the principal task-related effects [12]. Nevertheless, evidence from controlled neuromuscular tasks indicates that age and sex may influence force steadiness and intermuscular coherence in selected muscle pairs and frequency bands [13]. Age-related differences in lower-limb intermuscular beta coherence have also been reported during dynamic motor tasks [14]. These findings highlight the importance of ensuring comparable demographic characteristics between groups. In the present study, the professional and amateur groups had comparable age and sex distributions. In addition, sEMG amplitude was normalized to muscle-specific maximal voluntary contraction values to reduce inter-individual variability in raw signal amplitude. Because the present study was designed primarily to examine competitive-level differences, age- and sex-specific effects were not analyzed separately and should be examined in larger, appropriately powered samples.

Despite the increasing application of sensing technologies in sports performance analysis, previous investigations of tennis serving have mainly focused on kinematic characteristics, ball velocity, joint mechanics, or isolated muscle activation patterns [15]. Relatively few studies have utilized sensor-based approaches to examine how trunk and lower-limb muscles interact during the flat serve from a time–frequency neuromuscular coordination perspective [16]. Existing wearable-sensor research in tennis has primarily assessed upper-limb muscle activity, racket motion, grip-related characteristics, or vibration transmission rather than frequency-specific coupling between the trunk and lower limbs. To our knowledge, no previous study has systematically examined time–frequency intermuscular coherence across the complete combinations of seven lower-limb muscles and three trunk muscles during the flat tennis serve. This limitation restricts the understanding of why high-level players can achieve more accurate and stable serves than lower-level players. Comparing athletes with different competitive levels using synchronized wearable sEMG sensing and movement recording may help identify skill-related neuromuscular strategies and reveal how trunk–lower limb coordination contributes to superior flat-serve performance [17,18].

Therefore, the purpose of this study was to employ a sensor-based assessment framework combining surface electromyography sensing, integrated electromyographic analysis, and time–frequency intermuscular coherence analysis to compare serve-placement accuracy, muscle activation characteristics, and trunk–lower limb neuromuscular coordination during flat tennis serves between professional and amateur tennis players. We hypothesized that professional players would demonstrate higher serve-placement accuracy, distinct muscle activation strategies, and different time–frequency coherence patterns between trunk and lower-limb muscles. More specifically, intermuscular coherence was examined across 21 predefined combinations between seven lower-limb muscles and three trunk muscles within the α, β, and γ frequency bands. The principal novelty of this study lies in moving beyond conventional behavioral and individual-muscle outcomes to characterize localized, frequency-specific differences in dynamic trunk–lower limb neuromuscular coupling. This study provides a sensor-based approach for understanding skill-level differences in tennis serve performance and may contribute to the development of objective neuromuscular evaluation methods and serve-specific training strategies.

## 2. Methods

### 2.1. Participants

An a priori sample-size calculation was performed using G*Power software (version 3.1.9.7; Heinrich Heine University Düsseldorf, Düsseldorf, Germany) for a two-tailed independent-samples *t*-test, assuming a large effect size (Cohen’s d = 1.30), a significance level of α = 0.05, a statistical power of 0.80, and an equal allocation ratio between groups. The analysis indicated that a minimum of 20 participants were required. To account for an anticipated attrition or invalid-data rate of approximately 20%, the planned sample size was increased to 24 participants. Accordingly, this cross-sectional group-comparison study included 24 eligible tennis players (17 male and 7 female), all of whom completed the study and were included in the final analysis. Participants were allocated to a professional group (n = 12; 9 male and 3 female) or an amateur group (n = 12; 8 male and 4 female) according to predefined criteria based on competitive achievement, tennis-specific training experience, and weekly training volume. In accordance with the classification criteria adopted in previous research [19], the professional group comprised high-level players who had received systematic tennis-specific training for at least 5 years, trained for at least 10 h per week, and achieved a top-three placement in an officially organized provincial-level tennis competition. The amateur group comprised players who had at least 2 years of regular tennis training experience, trained for no more than 5 h per week, and could consistently perform the flat serve but had not achieved a top-three placement in a provincial-level competition or an equivalent competitive result. Players whose training or competitive characteristics did not clearly meet the criteria for either group were excluded to reduce within-group heterogeneity. All participants completed the testing tasks using their habitual racket-holding side. The basic characteristics of the two groups are presented in Table 1.

The exclusion criteria were as follows: (1) acute injury to the lower limbs, trunk, shoulder, elbow, or wrist within the past 6 months; (2) a history of severe muscular, skeletal, or joint disorders that could affect the performance of the flat serve; (3) neurological disorders, vestibular dysfunction, or other conditions that could affect motor control; (4) participation in high-intensity training or formal competition within 24 h before testing, or the presence of obvious fatigue during testing; and (5) abnormal electromyographic signal quality, a movement pattern that clearly failed to meet the technical requirements of the flat serve, or inability to complete the prescribed testing tasks during the experiment.

This study was approved by the Ethics Committee of Hangzhou Normal University and was conducted in accordance with the Declaration of Helsinki. Before formal testing, all participants were informed of the study purpose, testing procedures, precautions, and potential risks, and voluntarily signed written informed consent. To minimize the influence of external factors on neuromuscular function and motor performance, participants were instructed to avoid high-intensity training, official competition, alcohol consumption, and other behaviors that could affect the testing results within 24 h before testing. Compliance with these requirements was verified through a structured verbal interview conducted upon arrival at the testing venue. A researcher asked each participant about compliance with the pre-test requirements and documented the responses on a pre-test screening record form. If non-compliance or any condition potentially affecting test performance was identified, testing was postponed and rescheduled after the required restriction period had been satisfied. All participants included in the final analysis confirmed compliance with the pre-test requirements.

### 2.2. Experimental Design

The primary outcome measures included serve-placement accuracy, integrated electromyography (IEMG) of the target muscles, and time–frequency intermuscular coherence indices between trunk and lower limb muscle groups. By comparing differences in muscle activation levels and time–frequency intermuscular coherence between the professional and amateur groups under the same flat-serve task, this study aimed to characterize trunk–lower limb force-generation coordination during flat tennis serving in players of different competitive levels.

#### 2.2.1. Testing Instruments

Surface electromyographic signals during the flat tennis serve were recorded using a wireless surface electromyography system (Delsys Trigno Wireless EMG System, Delsys Inc., Natick, MA, USA). The sampling frequency was set at 2000 Hz, and the signal acquisition bandwidth was 20–450 Hz. During testing, this system was used to record surface electromyographic signals from the target lower limb and trunk muscles on the dominant or primary force-generating side, providing raw data for subsequent integrated electromyography and time–frequency intermuscular coherence analyses.

The serving movement was synchronously recorded using a high-speed camera system (Photron FASTCAM Mini AX200, Photron, Tokyo, Japan) at a recording frequency of 200 Hz. The high-speed camera was positioned laterally or oblique-laterally to the participant to capture the complete flat-serve sequence, including the preparation stance, ball toss, lower-limb knee flexion, propulsive extension, trunk rotation, racket–ball contact, and follow-through phases. The high-speed video data were used for offline frame-by-frame identification of key movement events and for subsequent segmentation of the serving movement cycle and movement phases.

To ensure temporal correspondence between surface electromyographic signals and serving movement events, an external TTL synchronization trigger system was used to synchronize the wireless surface electromyography system and the high-speed camera system. At the beginning of each valid serve trial, a TTL pulse was generated to simultaneously initiate electromyographic signal acquisition and high-speed video recording. During offline analysis, the TTL synchronization signal was used as the common temporal reference, and electromyographic signals within the corresponding movement phases were extracted based on the key events identified from the high-speed video recordings.

All tests were conducted on a standard tennis court using standard tennis balls and the prescribed serving area. According to the study purpose, a predefined target landing area was set within the opposite valid service box and clearly marked using boundary lines or marking tape. After each serve, whether the ball landed within the opposite valid service box and whether it hit the predefined target area were recorded to calculate serve-placement accuracy.

#### 2.2.2. Definition of the Flat Tennis Serve Technique

The technique analyzed in this study was the flat tennis serve, hereafter referred to as FTS. FTS is a typical form of the overhead tennis serve, characterized primarily by a forward-oriented ball strike, relatively limited ball spin, and an emphasis on ball speed, serve depth, and placement control. Compared with slice and topspin serves, FTS relies more strongly on the sequential kinetic-chain transfer among lower-limb extension, hip–trunk rotation, and upper-limb racket acceleration [20].

The FTS consists of a series of continuous movement phases, including the preparation stance, ball toss, lower-limb knee-flexion loading, propulsive lower-limb extension, hip–trunk rotation, upper-limb racket acceleration, racket–ball contact, and follow-through. This movement reflects a kinetic-chain force-generation pattern in which force is initiated from the lower limbs, transmitted through the hips and trunk, and ultimately delivered to the upper limb for ball striking.

During testing, all participants performed the task using their habitual racket-holding side and their stable FTS technique. To ensure consistency in serve type, participants were required to perform a conventional overhead flat serve and were not allowed to intentionally use slice serves, topspin serves, sidespin serves, underhand serves, or other serve techniques that clearly altered the technical structure of the flat serve [21].

Trials were excluded from subsequent analysis and repeated if movement interruption, a clear change in serve type, excessive spin serve, foot fault, invalid serve, abnormal electromyographic signals, or synchronization failure occurred during testing. Valid trials were repeatedly collected until the required number of FTS trials was completed.

#### 2.2.3. Selection of Target Muscles

Surface electromyographic signals were recorded from selected lower limb and trunk muscles on the dominant or primary force-generating side. The target lower limb muscles included the tibialis anterior (TA), lateral gastrocnemius (LG), soleus (SOL), vastus medialis (VM), vastus lateralis (VL), biceps femoris (BF), and gluteus maximus (GM). The target trunk muscles included the latissimus dorsi (LD), erector spinae (ES), and rectus abdominis (RA).

The selection of these muscles was based on the key functional demands of FTS, including lower-limb propulsion, hip extension, trunk stabilization, and trunk–upper-limb kinetic-chain force transfer. TA, LG, and SOL are primarily involved in ankle stabilization, foot support, and propulsive force generation; VM and VL mainly reflect knee extension control; BF and GM contribute to hip extension and posterior-chain force production; and LD, ES, and RA reflect trunk stabilization, trunk extension/flexion control, and force transfer between the trunk and upper limb. Together, these muscles cover the major functional components of trunk–lower limb force-generation coordination during FTS and were used to analyze muscle activation levels and time–frequency intermuscular coherence characteristics in tennis players of different competitive levels [22].

#### 2.2.4. Testing Procedures

After arriving at the testing venue, participants first completed basic information registration, and their physical condition and eligibility for testing were confirmed by the researchers. Participants then performed a standardized warm-up, including low-intensity jogging, dynamic stretching, lower limb and trunk activation exercises, shoulder, elbow, and wrist mobility exercises, and low-intensity tennis serve practice. The total warm-up duration was controlled within 10–15 min to ensure that participants entered a relatively stable state of motor readiness.

After the warm-up, the skin over each target muscle was prepared using a standardized procedure, including shaving, light abrasion of the stratum corneum, and cleaning with 75% alcohol to reduce skin impedance and improve electromyographic signal quality. Surface electromyography electrodes were placed over the muscle belly along the direction of the muscle fibers and secured with elastic tape to minimize electrode displacement and movement artifacts during FTS. All electrodes were placed by the same researcher to reduce the influence of operator-related variability on electromyographic signal acquisition.

After electrode placement, participants performed low-intensity practice serves, during which the researchers monitored the signal quality and wireless transmission status of each electromyographic channel in real time. Formal testing began only after stable signals, normal wireless transmission, and the absence of obvious power-line interference, movement artifacts, or electrode detachment were confirmed.

Before formal testing, participants completed 5–10 familiarization FTS trials to become accustomed to the testing environment, target landing area, and serving rhythm. Familiarization trials were not included in subsequent analyses. Participants then completed the formal FTS test. A 20–30 s interval was provided between consecutive serves to reduce fatigue effects caused by repeated serving. Surface electromyographic signals, high-speed video data, and serve-placement outcomes were recorded synchronously throughout the test. If movement interruption, a clear change in serve type, a foot fault, an invalid serve, abnormal electromyographic signals, or synchronization failure occurred, the trial was considered invalid and was repeated until the required number of valid FTS trials was completed.

### 2.3. Data Processing and Outcome Measures

#### 2.3.1. Movement Cycle and Phase Segmentation

To ensure consistent temporal boundaries and clear biomechanical meaning for surface electromyographic analysis, the movement cycle of the flat tennis serve (FTS) was segmented based on high-speed video data. The FTS analysis cycle was defined as the period from the onset of lower-limb knee flexion to racket–ball contact. The onset of lower-limb knee flexion was defined as the moment when the participant transitioned from the preparation stance into the knee-flexion loading phase, with the knee joint beginning to flex visibly or the body center of mass beginning to descend. Racket–ball contact was defined as the key instant when the racket contacted the tennis ball [23].

According to the movement structure and kinetic-chain transfer characteristics of FTS, the complete analysis cycle was further divided into three phases: the knee-flexion loading phase, propulsive extension phase, and pre-impact acceleration phase. The knee-flexion loading phase was defined as the period from lower-limb knee-flexion onset to maximum knee flexion or the lowest position of the body center of mass, primarily reflecting lower-limb flexion and loading. The propulsive extension phase was defined as the period from maximum knee flexion or the lowest position of the body center of mass to the substantial completion of lower-limb extension, primarily reflecting hip, knee, and ankle extension and upward body acceleration. The pre-impact acceleration phase was defined as the period from the substantial completion of lower-limb extension to racket–ball contact, primarily reflecting trunk rotation, forward-upward force transfer, and upper-limb racket acceleration.

All key movement events were identified offline frame by frame using high-speed video recordings. After the movement cycle and phase boundaries of each valid FTS trial were determined, surface electromyographic signals within the corresponding time windows were extracted for subsequent integrated electromyography and time–frequency intermuscular coherence analyses.

#### 2.3.2. Serve-Placement Accuracy

Serve-placement accuracy was used to evaluate FTS performance. All tests were conducted on a standard tennis court, and participants performed FTS trials within the prescribed serving area. According to the technical characteristics of FTS and the purpose of the study, a predefined target landing area was set within the opposite valid service box. The target area was in the rear half of the opposite service box near the center line to assess serve depth control and placement accuracy. The target area was uniformly marked before testing using boundary lines or marking tape to ensure consistency of testing conditions across participants.

During formal testing, each participant completed 10 valid FTS trials. A serve was considered valid if the ball passed over the net and landed within the opposite valid service box. If a valid serve also landed within the predefined target area, it was recorded as a target hit; if it landed within the valid service box but outside the target area, it was recorded as a target miss. Trials were considered invalid and repeated if the serve hit the net, landed out, involved a foot fault, was interrupted, clearly deviated from the technical requirements of FTS, or had synchronization failure, until each participant completed 10 valid FTS trials.

The number of target hits across the 10 valid FTS trials was recorded for each participant, and serve-placement accuracy was calculated as the percentage of target hits relative to the number of valid trials. Serve-placement accuracy was calculated as follows [24]:

$$Accuracy=\frac{N_{target}}{N_{valid}}\times 100\%$$

where $N_{target}$ represents the number of serves that landed within the target area, and $N_{valid}$ represents the number of valid FTS trials. In the present study, $N_{valid}=10$.

#### 2.3.3. Surface Electromyographic Signal Preprocessing and IEMG Calculation

Raw surface electromyographic signals were processed offline. First, the raw electromyographic signals were band-pass filtered at 20–400 Hz using a fourth-order zero-phase Butterworth filter to remove low-frequency movement artifacts and high-frequency noise. If obvious power-line interference was present, a 50-Hz notch filter was applied. Based on the key movement events identified from high-speed video recordings, electromyographic signals from each valid FTS trial were extracted from lower-limb knee-flexion onset to racket–ball contact and further segmented into the knee-flexion loading phase, propulsive extension phase, and pre-impact acceleration phase.

For IEMG analysis, the filtered electromyographic signals were full-wave rectified. To reduce the influence of inter-individual differences in skin impedance, subcutaneous fat thickness, and electrode placement on electromyographic amplitude, electromyographic signals were normalized to the maximal voluntary contraction (MVC) value. MVC testing for each target muscle was performed before the formal serve test, and the maximum electromyographic amplitude obtained during the stable contraction phase was used as the normalization reference.

Before the formal serve test, MVC testing was performed for each target muscle using standardized manual-resistance positions. For the tibialis anterior, participants performed maximal ankle dorsiflexion while seated with the ankle in a neutral position. The lateral gastrocnemius was tested during maximal plantar flexion with the knee extended, whereas the soleus was tested during seated plantar flexion with the knee flexed to approximately 90°. The vastus medialis and vastus lateralis were tested during seated knee extension with the knee flexed to approximately 60°. The biceps femoris was tested during prone knee flexion at approximately 45°, and the gluteus maximus was tested during prone hip extension with the knee flexed to approximately 90°. For the latissimus dorsi, participants performed resisted shoulder extension and adduction while seated. The erector spinae was tested during resisted trunk extension in the prone position, and the rectus abdominis was tested during resisted trunk flexion in the supine position. All tests were performed on the habitual racket-holding or primary force-generating side.

For each muscle, participants completed three 5-s MVC trials, with 60 s of rest between trials and at least 2 min between muscle tests to minimize fatigue. Standardized verbal encouragement was provided throughout each contraction. The first and last 1 s of each trial were excluded to reduce the influence of force-development and relaxation phases. The highest 500-ms root mean square EMG value obtained from the middle 3 s across the three valid trials was defined as the MVC reference value for that muscle. If the highest values from two trials differed by more than 10%, an additional trial was performed after sufficient rest. The same researcher administered all MVC tests and provided manual resistance to improve procedural consistency. Serve-related EMG amplitude was subsequently expressed relative to the corresponding muscle-specific MVC reference value (%MVC).

The normalized electromyographic signal was calculated as follows:

$$EMG_{norm}(t)=\frac{|EMG(t)|}{EMG_{MVC}}\times 100\%$$

where EMG(t) represents the electromyographic signal amplitude at time t, and $EMG_{MVC}$ represents the maximum electromyographic amplitude obtained from the corresponding muscle during MVC testing.

IEMG was used to quantify the overall activation level of the target muscles during the FTS movement cycle or specific movement phases. Normalized IEMG was calculated as follows [25]:

$$IEMG=\int_{t_{1}}^{t_{2}}EMG_{norm}(t)\;dt$$

where $t_{1}$ and $t_{2}$ represent the start and end times of the analyzed movement cycle or movement phase, respectively. IEMG values were expressed as %MVC⋅s. For each participant, IEMG values were averaged across the 10 valid FTS trials and used as the final IEMG value of the corresponding muscle within the analyzed movement cycle or phase.

#### 2.3.4. Time–Frequency Intermuscular Coherence Analysis

Time–frequency intermuscular coherence analysis was used to evaluate the common oscillatory characteristics between trunk and lower limb muscle groups across different time and frequency ranges during FTS, thereby reflecting neuromuscular coordination between trunk and lower limb muscles during rapid serving. In this study, time–frequency intermuscular coherence was calculated from preprocessed surface electromyographic signals based on an intermuscular coherence analysis framework.

First, raw surface electromyographic signals were band-pass filtered at 20–400 Hz and full-wave rectified. Based on the key movement events identified from high-speed video recordings, electromyographic signals from each valid FTS trial were extracted from lower-limb knee-flexion onset to racket–ball contact. To ensure a consistent time dimension across participants and trials, all valid FTS cycles were linearly time-normalized and interpolated to a 500-ms time series, with one data point corresponding to 1 ms. A 10-ms moving average window was then applied to obtain the electromyographic envelope for subsequent time–frequency coherence analysis [26].

This study focused on time–frequency intermuscular coherence between the trunk muscles and the major lower-limb muscles involved in postural control and force generation during FTS. Intermuscular coherence was analyzed for all pairwise combinations between seven lower-limb muscles—the tibialis anterior (TA), lateral gastrocnemius (LG), soleus (SOL), vastus medialis (VM), vastus lateralis (VL), biceps femoris (BF), and gluteus maximus (GM)—and three trunk muscles—the latissimus dorsi (LD), erector spinae (ES), and rectus abdominis (RA)—resulting in 21 predefined trunk–lower limb muscle pairs. Specifically, the analyzed pairs were TA–LD, LG–LD, SOL–LD, VM–LD, VL–LD, BF–LD, and GM–LD; TA–ES, LG–ES, SOL–ES, VM–ES, VL–ES, BF–ES, and GM–ES; and TA–RA, LG–RA, SOL–RA, VM–RA, VL–RA, BF–RA, and GM–RA. These muscle pairs were selected to comprehensively characterize functional coupling between lower-limb muscles involved in ankle control, postural support, knee extension, hip extension, and propulsion and trunk muscles involved in stabilization, trunk control, and proximal kinetic-chain coordination during the flat serve. The inclusion of all lower-limb–trunk combinations also avoided the selective analysis or reporting of only those muscle pairs showing significant between-group differences.

Time–frequency coherence was calculated using a short-time Fourier transform. Specifically, the electromyographic signals from two muscles were segmented into equal-length overlapping time windows using a Hamming window, with the window width set to 200 data points and the overlap set to 75%. Within each time window, the time–frequency distribution of the two electromyographic signals was estimated, and the autospectra and cross-spectrum of the two signals were obtained. To reduce the influence of random noise on coherence estimation, the autospectra and cross-spectrum were smoothed using two-dimensional convolution. Time–frequency intermuscular coherence was calculated from the smoothed cross-spectrum and autospectra as follows:

$$C_{xy}(f,t)=\frac{|S_{xy}(f,t){|}^{2}}{S_{xx}(f,t)S_{yy}(f,t)}$$

where $C_{xy}\left(f,t\right)\;$ represents the time–frequency coherence between muscle x and muscle y at frequency f and time t, $S_{xy}(f,t)$ represents the smoothed cross-spectrum of the two muscle signals, and $S_{xx}(f,t)$ and $S_{yy}(f,t)$ represent the smoothed autospectra of the two muscle signals, respectively. Coherence values range from 0 to 1, with higher values indicating stronger common oscillatory activity between the two muscles within the corresponding time–frequency range.

To determine whether coherence values were statistically significant, a confidence limit threshold was calculated based on the number of effective data segments used for coherence estimation. Coherence regions exceeding this threshold were considered to indicate significant intermuscular coherence. The confidence limit threshold was calculated as follows:

$$Thr=1-\alpha^{\frac{1}{L-1}}$$

where Thr represents the significance threshold for coherence, α represents the significance level, which was set at 0.05 in this study, and L represents the number of effective data segments used for coherence estimation.

To further reduce spurious coherence caused by signal amplitude characteristics or random phase relationships, a surrogate-data correction procedure was applied. Surrogate data were generated by randomizing the phase of the original electromyographic signal while preserving its amplitude spectrum. Time–frequency coherence was calculated separately for the original electromyographic signals and the surrogate data, and the difference between the original coherence and surrogate coherence was used as the corrected coherence result. When the original coherence was higher than the surrogate coherence, the difference was retained; when the difference was less than or equal to zero, the coherence value at that time–frequency point was set to zero. The surrogate-corrected coherence was calculated as follows:

$$C_{corr}(f,t)=max\left[C_{real}(f,t)-C_{surrogate}(f,t),0\right]$$

where $C_{corr}\left(f,t\right)\;$ represents the corrected time–frequency coherence, $C_{real}\left(f,t\right)\;$ represents the coherence calculated from the original electromyographic signals, and $C_{surrogate}(f,t)$ represents the coherence calculated from the surrogate data.

To quantify coherence strength within specific frequency bands and movement phases, the area of significant coherence was calculated. The area of significant coherence was defined as the integrated area of corrected coherence values exceeding the significance threshold within a specific movement phase and frequency band. It was calculated as follows [27]:

$$AZ=\sum_{t=t_{1}}^{t_{2}}\sum_{f=f_{1}}^{f_{2}}C_{corr}(f,t)\cdot \Delta t\cdot \Delta f$$

where AZ represents the area of significant coherence, $t_{1}$ and $t_{2}$ represent the start and end times of the analyzed movement phase, $f_{1}$ and $f_{2}$ represent the lower and upper limits of the analyzed frequency band, and Δt and Δf represent the time and frequency resolutions, respectively.

This study focused on intermuscular coherence in the α, β, and γ frequency bands. The α band was defined as 8–15 Hz, the β band as 15–30 Hz, and the γ band as 30–50 Hz. AZ values were extracted for each trunk–lower limb muscle pair within the knee-flexion loading phase, propulsive extension phase, and pre-impact acceleration phase, and were used to compare trunk–lower limb neuromuscular coordination characteristics during FTS between the professional and amateur groups.

### 2.4. Statistical Analysis

Statistical analyses were performed using SPSS 26.0 software. Continuous variables are presented as mean ± standard deviation. The Shapiro–Wilk test was used to assess the normality of each continuous variable, and Levene’s test was used to evaluate the homogeneity of variance between the professional and amateur groups.

Given the cross-sectional group-comparison design, two-tailed independent-samples *t*-tests were used to compare serve-placement accuracy, IEMG values of the target muscles, and the area of significant coherence (AZ) for the different trunk–lower limb muscle pairs between the professional and amateur groups.

To control the risk of false-positive findings arising from multiple comparisons, the Benjamini–Hochberg false discovery rate (BH–FDR) procedure was applied to predefined families of related outcomes. For the IEMG analysis, the raw *p*-values corresponding to the 10 target muscles were adjusted as one family. For the time–frequency intermuscular coherence analysis, the raw *p*-values corresponding to the 21 trunk–lower limb muscle pairs were adjusted separately within each frequency band. Both unadjusted *p*-values, reported as raw *p*-values, and FDR-adjusted *p*-values, reported as FDR q-values, are presented in the corresponding tables and appendix. For outcome families involving multiple comparisons, statistical significance was determined at FDR q < 0.05. Raw *p*-values are reported for transparency but were not used to determine statistical significance after FDR correction.

Effect sizes were reported as Cohen’s d and calculated as the standardized difference between the professional and amateur group means using the pooled standard deviation. The direction of all t-values and Cohen’s d values was consistently defined as the professional group minus the amateur group. Accordingly, positive t-values and Cohen’s d values indicate higher values in the professional group, whereas negative values indicate higher values in the amateur group.

For single outcomes not involving multiple comparisons, such as serve-placement accuracy, no FDR correction was applied, and statistical significance was determined using a two-tailed raw *p*-value of <0.05. All reported tests were two-tailed. Results are presented as t-values, raw *p*-values, FDR q-values where applicable, and Cohen’s d values [28].

## 3. Results

### 3.1. Comparison of Serve-Placement Accuracy

As shown in Figure 1 and Table 2, serve-placement accuracy differed significantly between the professional and amateur groups. The professional group demonstrated significantly higher serve-placement accuracy than the amateur group (t = 6.26, *p* < 0.001, Cohen’s d = 2.55).

### 3.2. Comparison of Muscle Activation Levels During FTS

As shown in Figure 2 and Table 3, significant between-group differences in IEMG were observed in selected target muscles during FTS after Benjamini–Hochberg FDR correction. The professional group showed significantly higher IEMG values than the amateur group in SOL (t = 3.12, raw *p* = 0.005, FDR q = 0.019, Cohen’s d = 1.27), VM (t = 2.92, raw *p* = 0.008, FDR q = 0.020, Cohen’s d = 1.19), VL (t = 2.60, raw *p* = 0.016, FDR q = 0.027, Cohen’s d = 1.06), GM (t = 3.17, raw *p* = 0.004, FDR q = 0.019, Cohen’s d = 1.29), and LD (t = 2.74, raw *p* = 0.012, FDR q = 0.024, Cohen’s d = 1.12). In contrast, the professional group showed a significantly lower IEMG value in RA than the amateur group (t = −3.07, raw *p* = 0.006, FDR q = 0.019, Cohen’s d = −1.25). No significant between-group differences were observed in TA, LG, BF, or ES after FDR correction (all FDR q > 0.05).

### 3.3. Comparison of Trunk–Lower Limb Time–Frequency Intermuscular Coherence During FTS

As shown in Figure 3 and Table S1, several trunk–lower limb muscle pair–frequency combinations showed nominal between-group differences in AZ values based on the unadjusted *p*-values. However, none of these differences remained statistically significant after the Benjamini–Hochberg false discovery rate correction was applied separately to the 21 muscle-pair comparisons within each frequency band (all FDR q > 0.05).

For the LD-related muscle pairs, nominal between-group differences were observed for TA–LD in the α band (t = 2.63, raw *p* = 0.015, FDR q = 0.100, Cohen’s d = 1.07), LG–LD in the α band (t = 2.38, raw *p* = 0.026, FDR q = 0.109, Cohen’s d = 0.97), and GM–LD in the α band (t = −3.26, raw *p* = 0.004, FDR q = 0.084, Cohen’s d = −1.33). The positive effect sizes for TA–LD and LG–LD indicated higher AZ values in the professional group, whereas the negative effect size for GM–LD indicated a higher AZ value in the amateur group. Nevertheless, none of these comparisons met the predefined FDR-adjusted significance criterion.

For the ES-related muscle pairs, LG–ES in the α band showed a nominal between-group difference (t = 2.54, raw *p* = 0.019, FDR q = 0.100, Cohen’s d = 1.04), with a higher AZ value in the professional group. TA–ES and LG–ES in the γ band each had a raw *p*-value of 0.050, but neither comparison was statistically significant after FDR correction (both FDR q = 0.245). No ES-related muscle pair–frequency combination reached the FDR-adjusted significance threshold.

For the RA-related muscle pairs, nominal between-group differences were observed for TA–RA in the α band (t = 2.57, raw *p* = 0.017, FDR q = 0.100, Cohen’s d = 1.05), β band (t = 2.09, raw *p* = 0.049, FDR q = 0.243, Cohen’s d = 0.85), and γ band (t = 2.13, raw *p* = 0.044, FDR q = 0.245, Cohen’s d = 0.87). Nominal differences were also observed for LG–RA in the α band (t = 2.09, raw *p* = 0.048, FDR q = 0.133, Cohen’s d = 0.85) and β band (t = 2.40, raw *p* = 0.025, FDR q = 0.243, Cohen’s d = 0.98), as well as for GM–RA in the α band (t = 2.17, raw *p* = 0.041, FDR q = 0.133, Cohen’s d = 0.89). All corresponding positive effect sizes indicated higher AZ values in the professional group. However, none of these comparisons remained statistically significant after FDR correction.

Overall, 10 muscle pair–frequency combinations across seven trunk–lower limb muscle pairs—TA–LD, LG–LD, GM–LD, LG–ES, TA–RA, LG–RA, and GM–RA—showed nominal differences based on raw *p* < 0.05. These nominal effects were concentrated primarily in the α band and were accompanied by moderate-to-large effect sizes. Nevertheless, all FDR-adjusted q-values exceeded 0.05, indicating that the observed between-group differences did not withstand correction for multiple comparisons. Therefore, these findings should be interpreted as exploratory patterns that may warrant confirmation in larger, independently powered studies rather than as definitive evidence of competitive-level differences in trunk–lower limb intermuscular coherence.

## 4. Discussion

The present study found that serve-placement accuracy was significantly higher in the professional group than in the amateur group during the flat tennis serve. Although this difference was expected, it confirms a behavioral distinction between the two competitive levels under standardized target conditions. This finding is consistent with previous studies showing that high-level tennis players demonstrate greater serve accuracy and more stable technical execution than lower-level players [29]. Serve accuracy depends not only on racket–ball contact but also on the organization of the preceding whole-body movement sequence [30], including ball-toss control, body alignment, and pre-impact posture [31]. Conversely, the lower accuracy observed in amateur players may reflect greater variability in coordinating lower-limb loading, trunk motion, and upper-limb action [32]. In the present study, serve-placement accuracy therefore served primarily as a behavioral reference for interpreting differences in neuromuscular function between competitive levels [33].

The muscle-activation results provide further insight into the neuromuscular strategies underlying flat-serve performance. After Benjamini–Hochberg FDR correction, professional players showed significantly greater IEMG values in SOL, VM, VL, GM, and LD, whereas RA activation was significantly greater in amateur players. Greater activation of the plantar flexor, knee extensors, hip extensor, and LD in professional players may indicate more pronounced recruitment of muscles involved in lower-limb support, propulsion, proximal stabilization, and coordination between the trunk and serving-side upper limb. In contrast, greater RA activation in amateur players may reflect increased reliance on anterior trunk activation to maintain stability during the serve. These findings suggest that competitive-level differences are related not only to the magnitude of muscle activation but also to its distribution across functionally distinct lower-limb and trunk muscles. However, because joint kinetics and ground reaction forces were not measured, these activation patterns should not be interpreted as direct evidence of mechanical force production or energy transfer.

In contrast to the IEMG findings, none of the time–frequency intermuscular coherence comparisons remained statistically significant after BH–FDR correction. Several muscle pair–frequency combinations showed nominal differences based on raw *p*-values, primarily in the α band and involving TA–LD, LG–LD, GM–LD, LG–ES, TA–RA, LG–RA, and GM–RA. Additional nominal differences were observed for TA–RA and LG–RA in the β band and for TA–RA in the γ band. Although these comparisons were accompanied by moderate-to-large effect sizes, all corresponding FDR q-values exceeded 0.05. Therefore, these findings cannot be regarded as confirmatory evidence of competitive-level differences in trunk–lower limb intermuscular coherence. Instead, they represent exploratory patterns that may help identify muscle pair–frequency combinations for hypothesis-driven investigation in larger samples. The concentration of the nominal effects in selected combinations, rather than across all muscles and frequencies, is broadly consistent with the possibility that expertise is associated with functionally selective organization of multi-muscle control [34]; nevertheless, this interpretation requires independent confirmation.

Among the LD-related muscle pairs, nominal raw-P differences suggested higher α-band AZ values for TA–LD and LG–LD in professional players, whereas GM–LD showed a higher α-band AZ value in amateur players. TA and LG contribute to ankle control and postural support, while LD contributes to trunk stabilization and coordination with the serving-side upper limb. These patterns could tentatively reflect differences in how distal lower-limb and hip-extensor activity is coupled with LD during the serve. However, none of these comparisons remained significant after FDR correction. Accordingly, the findings should not be interpreted as demonstrating stronger or more effective shared neural input in either group. Moreover, higher coherence does not automatically indicate superior coordination because it may reflect efficient common neural drive, task-specific coactivation, or reduced independence between muscles. The opposite directions observed across the nominal LD-related effects further argue against interpreting skilled performance as being characterized by a generalized increase in intermuscular coherence.

For the ES-related muscle pairs, LG–ES in the α band showed a nominally higher AZ value in professional players, but this difference did not remain significant after FDR correction. Thus, although the unadjusted result may suggest a potential relationship between plantar-flexor support and trunk-extensor control, it cannot establish a reliable competitive-level difference. Similarly, the nominal RA-related patterns suggested higher AZ values in professional players for TA–RA across the α, β, and γ bands; LG–RA across the α and β bands; and GM–RA in the α band. These patterns may tentatively indicate differences in the coupling of ankle-control, plantar-flexor, and hip-extensor muscles with anterior trunk activation. Nevertheless, because all corresponding FDR q-values exceeded 0.05, these observations should be treated as hypothesis-generating rather than as evidence that professional players organize trunk–lower limb coupling more effectively or more selectively. Larger studies are required to determine whether these muscle pair–frequency patterns are reproducible and meaningfully related to flat-serve performance [35].

The predominance of nominal raw-P effects in the α band may suggest a possible contribution of lower-frequency common neural input associated with postural regulation and sensorimotor integration. The additional nominal β- and γ-band effects in selected RA-related pairs may similarly point to more rapid or task-dependent components of shared neural drive. However, these interpretations must be considered cautiously because none of the frequency-specific effects survived FDR correction. Furthermore, individual frequency bands should not be assigned exclusively to specific neural mechanisms, particularly because intermuscular coherence reflects statistical coupling between muscle signals rather than a direct measurement of central neural activity. Its functional interpretation depends on the muscles involved, movement phase, frequency band, task demands, and direction of the observed group differences [36].

Compared with assessments based solely on behavioral performance or EMG amplitude, time–frequency intermuscular coherence can provide complementary information regarding the frequency-specific organization of common oscillatory activity between functionally related muscles. In the present study, however, the strongest evidence for competitive-level differences was provided by serve-placement accuracy and FDR-corrected IEMG outcomes, rather than by the coherence results. The nominal coherence patterns may nevertheless inform the selection of specific muscle pairs and frequency bands for future confirmatory research. Accordingly, the principal contribution of the present study lies in demonstrating distinct distributions of trunk and lower-limb muscle activation between professional and amateur players while providing preliminary, exploratory evidence concerning potential differences in frequency-specific intermuscular coupling. These results support the view that skilled flat-serve performance may involve selective neuromuscular organization rather than uniformly increased muscle activation or coherence [37]. However, the coherence findings should not be interpreted as definitive evidence of competitive-level differences, and neither IEMG nor coherence provides direct mechanical evidence of force or energy transmission.

### 4.1. Practical Implications

The findings of this study provide practical implications for tennis coaching and performance enhancement. The higher activation of the soleus (SOL), vastus medialis (VM), vastus lateralis (VL), gluteus maximus (GM), and latissimus dorsi (LD) in professional players suggests that effective flat-serve training should emphasize lower-limb propulsion, hip extension, and trunk–upper-limb force transfer rather than focusing only on racket movement. Therefore, coaches may incorporate lower-limb strength training, plyometric exercises, and serve-specific coordination drills to improve kinetic-chain efficiency. In addition, the distinct trunk–lower limb intermuscular coherence patterns indicate that training should focus on the timing and coordination of force generation among muscle groups, rather than simply increasing muscle activation levels.

### 4.2. Limitations

Several limitations should be acknowledged. First, the relatively small, male-only sample may limit the generalizability of the findings. Second, the cross-sectional design precludes causal conclusions regarding training-related neuromuscular adaptations. Third, sEMG recordings were limited to selected dominant-side trunk and lower-limb muscles, while IEMG and intermuscular coherence cannot fully characterize mechanical sequencing, force transmission, or central neural mechanisms. In addition, the high-speed camera was used only for movement-event identification and serve-cycle segmentation, without quantifying joint kinematics or ground reaction forces; therefore, the findings reflect neuromuscular coordination associated with kinetic-chain function rather than direct mechanical evidence of force or energy transfer. Finally, serve performance was assessed primarily through placement accuracy, without measuring ball or racket-head velocity, valid-serve percentage, or between-trial consistency. Future studies should include larger and more diverse samples and integrate longitudinal designs, three-dimensional kinematics, IMUs, force platforms, muscle activation onset, and muscle synergy analyses to provide a more comprehensive evaluation of serve mechanics and neuromuscular coordination.

## 5. Conclusions

This study found that professional tennis players achieved significantly higher flat-serve placement accuracy than amateur players. After Benjamini–Hochberg FDR correction, professional players also showed significantly higher IEMG values in the soleus, vastus medialis, vastus lateralis, gluteus maximus, and latissimus dorsi, but significantly lower rectus abdominis activation. Although several trunk–lower limb muscle pair–frequency combinations showed nominal differences based on raw *p*-values and moderate-to-large effect sizes, none remained statistically significant after FDR correction. Therefore, the time–frequency intermuscular coherence findings should be considered exploratory and require confirmation in larger studies. Overall, the results suggest that competitive-level differences in flat-serve performance are associated primarily with serve-placement accuracy and the distribution of trunk and lower-limb muscle activation. Training programs may emphasize lower-limb support and propulsion, hip extension, and trunk stabilization rather than focusing exclusively on arm or racket action. Time–frequency intermuscular coherence remains a potentially useful method for investigating neuromuscular coordination during tennis serving, but its ability to distinguish skill levels was not conclusively demonstrated in the present sample.

## Figures and Tables

**Figure 1:**
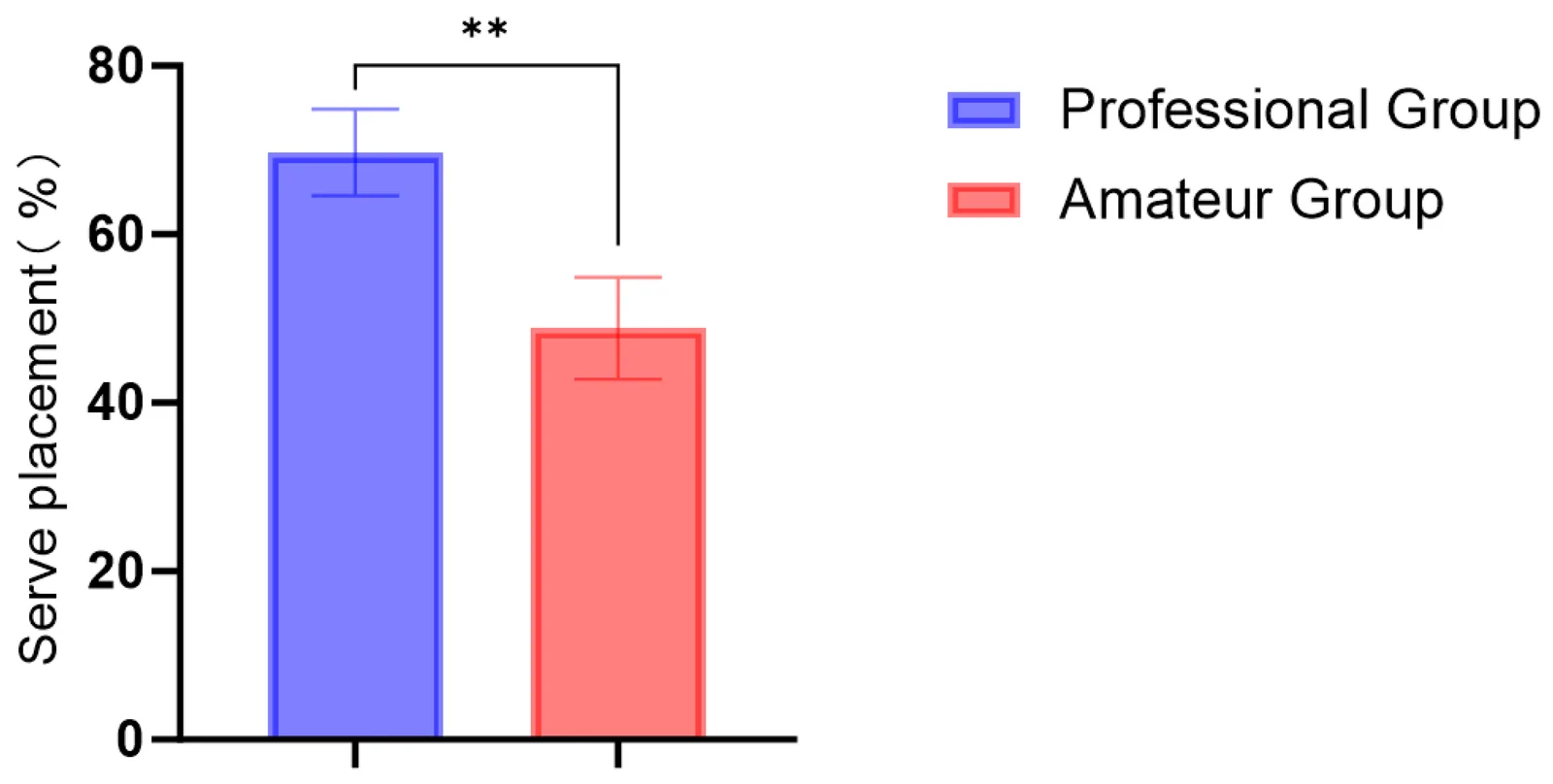
Comparison of serve-placement accuracy between the professional and amateur groups. ** Indicates *p* < 0.01.

**Figure 2:**
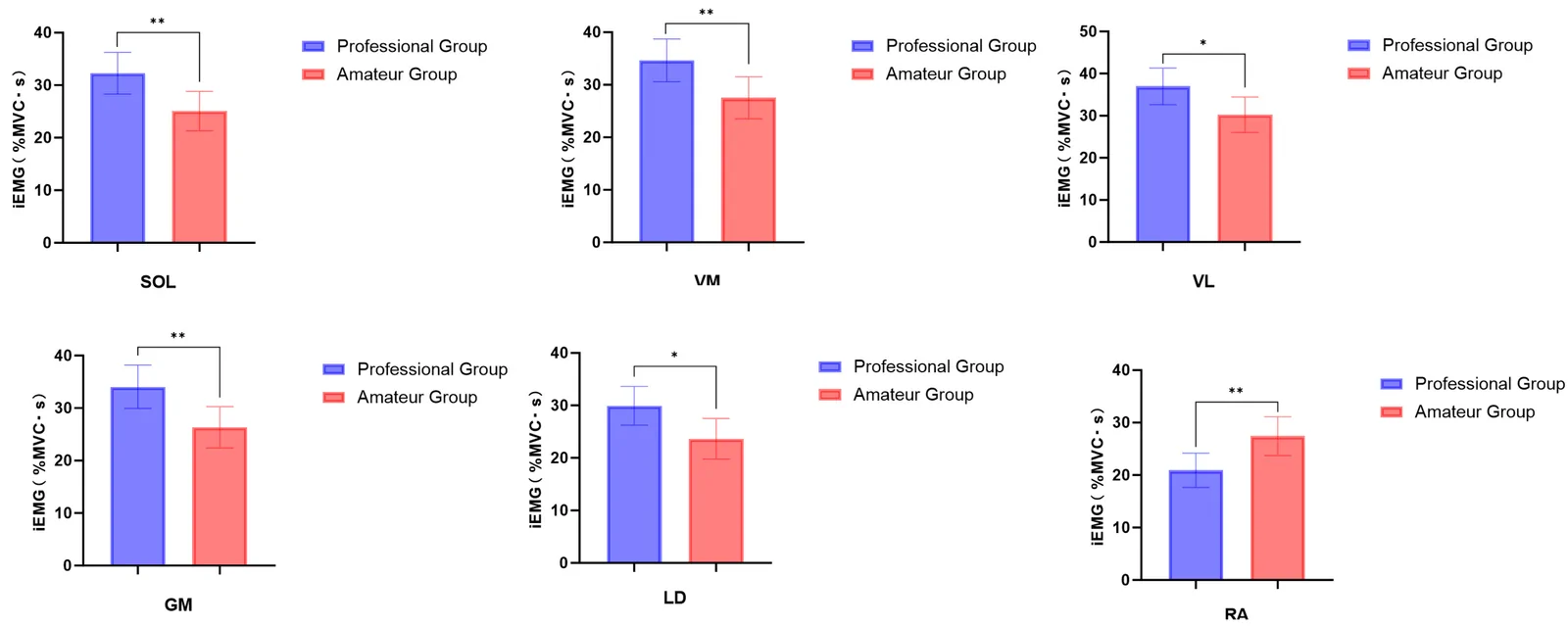
Comparison of iEMG values of target muscles during FTS between the professional and amateur groups. * indicates *p* < 0.05 and ** indicates *p* < 0.01.

**Figure 3:**
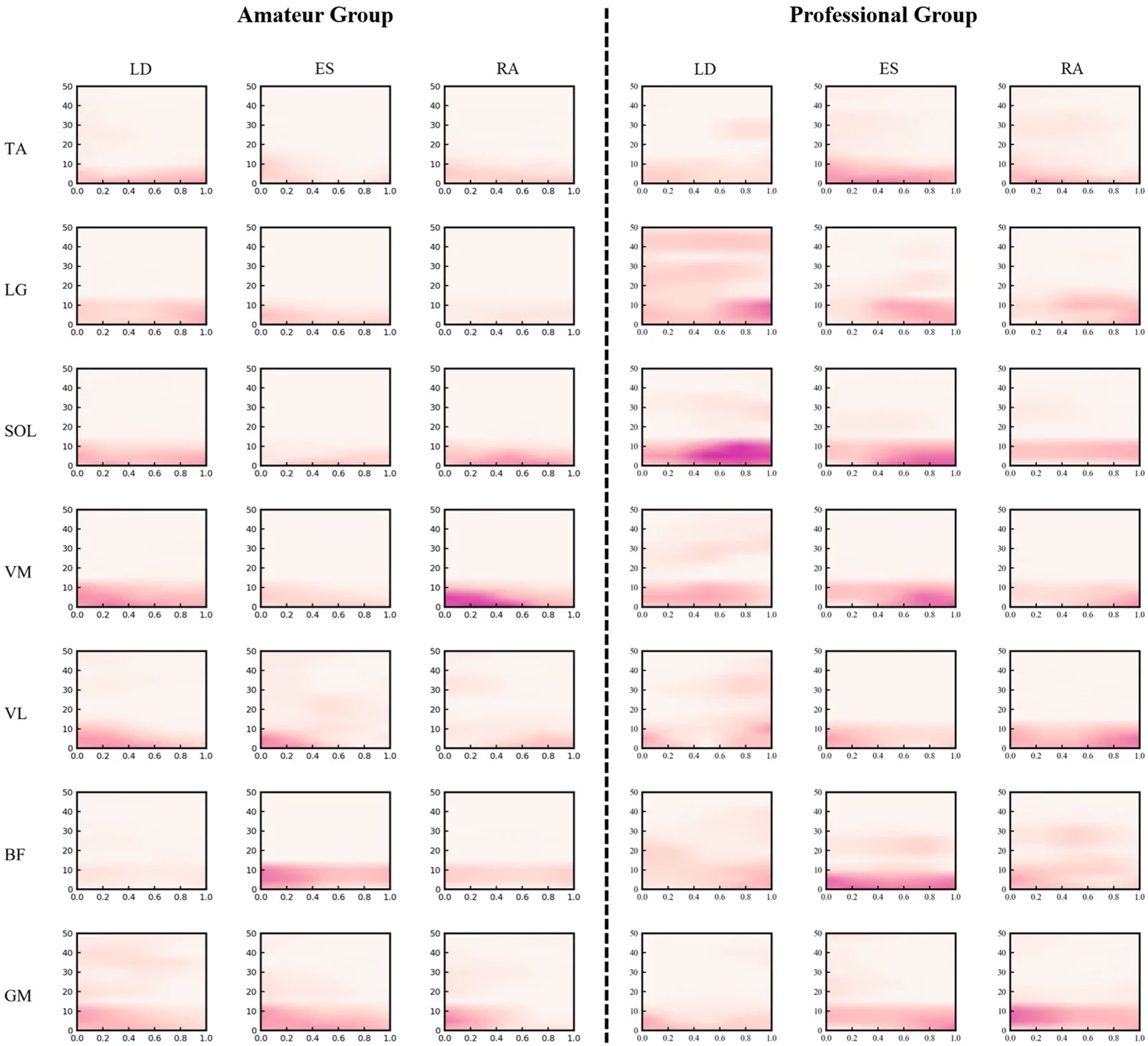
Comparison of trunk–lower limb time–frequency intermuscular coherence during FTS between the professional and amateur groups.

**Table 1:** Comparison of participant characteristics between the professional and amateur groups.

Variable	Professional Group (n = 12)	Amateur Group (n = 12)	t	*p*	Cohen’s d
Age (years)	22.3 ± 2.0	21.7 ± 1.8	0.77	0.449	0.32
Height (cm)	180.8 ± 5.6	178.9 ± 6.0	0.80	0.431	0.33
Body mass (kg)	73.6 ± 7.1	70.8 ± 6.8	0.99	0.335	0.40
BMI (kg/m^2^)	22.5 ± 1.7	22.1 ± 1.9	0.54	0.594	0.22
Sex, male/female (*n*)	9/3	8/4	—	—	—

**Table 2:** Comparison of serve-placement accuracy between the professional and amateur groups.

Variable	Professional Group	Amateur Group	t	*p*	Cohen’s d
Serve-placement accuracy (%)	69.2 ± 7.4	48.3 ± 8.9	6.26	<0.001	2.55

Note: Data are presented as mean ± standard deviation. The between-group difference was examined using a two-tailed independent-samples *t*-test, with 12 participants in each group. Because serve-placement accuracy was analyzed as a single behavioral outcome, no FDR correction was required. The t-value and Cohen’s d were calculated as the professional group minus the amateur group; therefore, positive values indicate higher values in the professional group, whereas negative values indicate higher values in the amateur group.

**Table 3:** Comparison of iEMG values of target muscles during FTS between the professional and amateur groups.

Muscle	Professional Group	Amateur Group	t	Raw *p*	FDR-Adjusted *p*	Cohen’s d
TA	18.6 ± 4.2	21.3 ± 4.8	−1.47	0.157	0.224	−0.60
LG	26.4 ± 5.1	23.7 ± 5.3	1.27	0.217	0.271	0.52
SOL	31.8 ± 5.8	24.6 ± 5.5	3.12	0.005	0.019	1.27
VM	34.2 ± 6.0	27.1 ± 5.9	2.92	0.008	0.020	1.19
VL	36.5 ± 6.4	29.8 ± 6.2	2.60	0.016	0.027	1.06
BF	22.8 ± 4.9	21.4 ± 5.0	0.69	0.496	0.496	0.28
GM	33.6 ± 6.1	25.9 ± 5.8	3.17	0.004	0.019	1.29
LD	29.4 ± 5.4	23.2 ± 5.7	2.74	0.012	0.024	1.12
ES	28.7 ± 6.3	31.5 ± 6.7	−1.05	0.303	0.337	−0.43
RA	20.5 ± 4.8	26.9 ± 5.4	−3.07	0.006	0.019	−1.25

Note: iEMG values are expressed as %MVC·s. Data are presented as mean ± standard deviation. Between-group differences were compared using independent-samples *t*-tests. Cohen’s d was calculated as the difference between the professional and amateur groups; positive values indicate higher values in the professional group, whereas negative values indicate higher values in the amateur group. TA, tibialis anterior; LG, lateral gastrocnemius; SOL, soleus; VM, vastus medialis; VL, vastus lateralis; BF, biceps femoris; GM, gluteus maximus; LD, latissimus dorsi; ES, erector spinae; RA, rectus abdominis.

## Data Availability

All data supporting the findings of this study are available within the main manuscript and its Supplementary Materials.

## Supplementary Materials

The following supporting information can be downloaded at: https://www.mdpi.com/article/10.3390/s26216899/s1.
