# Supplementary material for: Sensor-Based Assessment of Trunk–Lower Limb Neuromuscular Coordination During Flat Tennis Serve Using Surface Electromyography and Time–Frequency Intermuscular Coherence Analysis
Source: Sensors (Basel). 2026 Oct 31;26(21):6899. doi: 10.3390/s26216899 (PMC13766367; doi:10.3390/s26216899)
Supplement: Supplementary file 1 [file sensors-26-06899-s001.zip › sensors-4511352-supplementary.pdf]

Table S1. Trunk–Lower Limb Time–Frequency Intermuscular Coherence During the Flat Tennis Serve

LD-related muscle pairs

Muscle pair	Frequency band	Professional group AZ (mean ± SD)	Amateur group AZ (mean ± SD)	t	Raw P	FDR q	Cohen's d
TA–LD	α	0.018 ± 0.018	0.004 ± 0.004	2.63	0.015	0.100	1.07
TA–LD	β	0.044 ± 0.056	0.015 ± 0.015	1.73	0.097	0.243	0.71
TA–LD	γ	0.024 ± 0.035	0.010 ± 0.022	1.17	0.253	0.332	0.48
LG–LD	α	0.021 ± 0.029	0.001 ± 0.002	2.38	0.026	0.109	0.97
LG–LD	β	0.040 ± 0.073	0.000 ± 0.000	1.90	0.071	0.243	0.77
LG–LD	γ	0.056 ± 0.118	0.000 ± 0.000	1.64	0.114	0.252	0.67
SOL–LD	α	0.006 ± 0.012	0.002 ± 0.004	1.10	0.285	0.374	0.45
SOL–LD	β	0.024 ± 0.050	0.003 ± 0.004	1.45	0.161	0.290	0.59
SOL–LD	γ	0.011 ± 0.020	0.003 ± 0.007	1.31	0.204	0.306	0.53
VM–LD	α	0.005 ± 0.011	0.007 ± 0.009	−0.49	0.631	0.693	−0.20
VM–LD	β	0.029 ± 0.055	0.002 ± 0.003	1.70	0.104	0.243	0.69
VM–LD	γ	0.011 ± 0.020	0.000 ± 0.000	1.91	0.070	0.245	0.78
VL–LD	α	0.011 ± 0.029	0.015 ± 0.022	−0.38	0.707	0.707	−0.16
VL–LD	β	0.015 ± 0.048	0.005 ± 0.010	0.71	0.487	0.511	0.29
VL–LD	γ	0.005 ± 0.016	0.022 ± 0.053	−1.06	0.299	0.348	−0.43
BF–LD	α	0.020 ± 0.053	0.004 ± 0.005	1.04	0.309	0.382	0.43
BF–LD	β	0.023 ± 0.057	0.006 ± 0.006	1.03	0.315	0.348	0.42
BF–LD	γ	0.012 ± 0.037	0.001 ± 0.002	1.03	0.315	0.348	0.42
GM–LD	α	0.006 ± 0.011	0.037 ± 0.031	−3.26	0.004	0.084	−1.33
GM–LD	β	0.010 ± 0.026	0.044 ± 0.059	−1.83	0.081	0.243	−0.75
GM–LD	γ	0.016 ± 0.046	0.047 ± 0.068	−1.31	0.204	0.306	−0.53

ES-related muscle pairs

Muscle pair	Frequency band	Professional group AZ (mean ± SD)	Amateur group AZ (mean ± SD)	t	Raw P	FDR q	Cohen's d

TA–ES	α	0.034 ± 0.057	0.007 ± 0.012	1.61	0.123	0.215	0.66
TA–ES	β	0.043 ± 0.082	0.001 ± 0.002	1.77	0.090	0.243	0.72
TA–ES	γ	0.015 ± 0.025	0.000 ± 0.000	2.08	0.050	0.245	0.85
LG–ES	α	0.022 ± 0.030	0.000 ± 0.000	2.54	0.019	0.100	1.04
LG–ES	β	0.020 ± 0.036	0.000 ± 0.000	1.92	0.067	0.243	0.79
LG–ES	γ	0.006 ± 0.010	0.000 ± 0.000	2.08	0.050	0.245	0.85
SOL–ES	α	0.004 ± 0.006	0.001 ± 0.003	1.55	0.136	0.220	0.63
SOL–ES	β	0.008 ± 0.019	0.000 ± 0.000	1.46	0.159	0.290	0.60
SOL–ES	γ	0.002 ± 0.004	0.000 ± 0.000	1.73	0.097	0.252	0.71
VM–ES	α	0.011 ± 0.019	0.000 ± 0.000	2.01	0.057	0.133	0.82
VM–ES	β	0.012 ± 0.032	0.000 ± 0.000	1.30	0.207	0.290	0.53
VM–ES	γ	0.004 ± 0.011	0.000 ± 0.000	1.26	0.221	0.309	0.51
VL–ES	α	0.005 ± 0.013	0.023 ± 0.055	−1.10	0.282	0.374	−0.45
VL–ES	β	0.000 ± 0.000	0.024 ± 0.059	−1.41	0.173	0.290	−0.58
VL–ES	γ	0.000 ± 0.000	0.022 ± 0.054	−1.41	0.172	0.306	−0.58
BF–ES	α	0.019 ± 0.032	0.000 ± 0.000	2.06	0.052	0.133	0.84
BF–ES	β	0.018 ± 0.048	0.000 ± 0.000	1.30	0.207	0.290	0.53
BF–ES	γ	0.000 ± 0.000	0.000 ± 0.000	1.92	0.068	0.245	0.78
GM–ES	α	0.024 ± 0.039	0.015 ± 0.021	0.70	0.489	0.571	0.29
GM–ES	β	0.010 ± 0.027	0.016 ± 0.019	−0.63	0.535	0.535	−0.26
GM–ES	γ	0.023 ± 0.052	0.006 ± 0.007	1.12	0.274	0.338	0.46

RA-related muscle pairs

**Muscle pair**	**Frequency band**	**Professional group AZ (mean ± SD)**	**Amateur group AZ (mean ± SD)**	**t**	**Raw P**	**FDR q**	**Cohen’s d**
TA–RA	α	0.024 ± 0.026	0.004 ± 0.007	2.57	0.017	0.100	1.05
TA–RA	β	0.034 ± 0.053	0.002 ± 0.003	2.09	0.049	0.243	0.85
TA–RA	γ	0.016 ± 0.026	0.000 ± 0.000	2.13	0.044	0.245	0.87

Muscle pair	Frequency band	Professional group AZ (mean ± SD)	Amateur group AZ (mean ± SD)	t	Raw P	FDR q	Cohen's d
LG–RA	α	0.024 ± 0.038	0.001 ± 0.003	2.09	0.048	0.133	0.85
LG–RA	β	0.009 ± 0.013	0.000 ± 0.000	2.40	0.025	0.243	0.98
LG–RA	γ	0.004 ± 0.007	0.000 ± 0.000	1.98	0.060	0.245	0.81
SOL–RA	α	0.005 ± 0.008	0.002 ± 0.005	1.10	0.283	0.374	0.45
SOL–RA	β	0.015 ± 0.028	0.000 ± 0.000	1.86	0.077	0.243	0.76
SOL–RA	γ	0.012 ± 0.030	0.000 ± 0.000	1.39	0.180	0.306	0.57
VM–RA	α	0.016 ± 0.032	0.000 ± 0.000	1.73	0.097	0.185	0.71
VM–RA	β	0.009 ± 0.023	0.000 ± 0.000	1.36	0.189	0.290	0.55
VM–RA	γ	0.021 ± 0.045	0.000 ± 0.000	1.62	0.120	0.252	0.66
VL–RA	α	0.006 ± 0.012	0.009 ± 0.020	−0.45	0.660	0.693	−0.18
VL–RA	β	0.003 ± 0.005	0.013 ± 0.032	−1.07	0.296	0.345	−0.44
VL–RA	γ	0.003 ± 0.009	0.008 ± 0.019	−0.82	0.419	0.440	−0.34
BF–RA	α	0.023 ± 0.045	0.000 ± 0.000	1.77	0.090	0.185	0.72
BF–RA	β	0.022 ± 0.052	0.004 ± 0.010	1.18	0.252	0.311	0.48
BF–RA	γ	0.003 ± 0.006	0.000 ± 0.000	1.73	0.097	0.252	0.71
GM–RA	α	0.027 ± 0.030	0.007 ± 0.011	2.17	0.041	0.133	0.89
GM–RA	β	0.010 ± 0.017	0.022 ± 0.030	−1.21	0.241	0.311	−0.49
GM–RA	γ	0.019 ± 0.031	0.013 ± 0.020	0.56	0.579	0.579	0.23

Revised note

Note: Values are presented as mean ± SD. Between-group comparisons were performed using two-tailed independent-samples t-tests, with 12 participants in each group. The t-values and Cohen's d values were calculated as the professional group minus the amateur group. Accordingly, positive t and Cohen's d values indicate higher values in the professional group, whereas negative values indicate higher values in the amateur group. Raw P represents the unadjusted two-tailed P-value. The Benjamini–Hochberg false discovery rate procedure was applied separately to the 21 muscle-pair comparisons within each frequency band, and the resulting adjusted P-values are reported as FDR q-values. Statistical significance was determined at FDR $q < 0.05$. AZ, area of significant coherence; TA, tibialis anterior; LG, lateral gastrocnemius; SOL, soleus; VM, vastus medialis; VL, vastus lateralis; BF, biceps femoris; GM, gluteus maximus; LD, latissimus dorsi; ES, erector spinae; RA, rectus abdominis.
